# Iron oxide nanoparticles can cross plasma membranes

**DOI:** 10.1038/s41598-017-11535-z

**Published:** 2017-09-12

**Authors:** Daniele Zanella, Elena Bossi, Rosalba Gornati, Carlos Bastos, Nuno Faria, Giovanni Bernardini

**Affiliations:** 1 Department of Biotechnology and Life Sciences, University of Insubria; Via Dunant 3, I-21100 Varese, Italy; 2 Interuniversity Center “The Protein Factory”, Politecnico di Milano and Università dell’Insubria, Via Mancinelli 7, I-20131 Milan, Italy; 3 Department of Veterinary Medicine, University of Cambridge, Madingley Road, Cambridge, CB3 OES UK

## Abstract

Iron deficiency is a major global public health problem despite decades of efforts with iron supplementation and fortification. The issue lies on the poor tolerability of the standard of care soluble iron salts, leading to non-compliance and ineffective correction of iron-deficiency anaemia. Iron nanoformulations have been proposed to fortify food and feed to address these issues. Since it was just postulated that some nanoparticles (NPs) might cross the plasma membrane also by a non-endocytotic pathway gaining direct access to the cytoplasm, we have studied iron NP uptake under this perspective. To this aim, we have used a recently tested protocol that has proven to be capable of following the cytoplasmic changes of iron concentration dynamics and we have demonstrated that iron oxide NPs, but not zerovalent iron NPs nor iron oxide NPs that were surrounded by a protein corona, can cross plasma membranes. By electrophysiology, we have also shown that a small and transient increase of membrane conductance parallels NP crossing of plasma membrane.

## Introduction

Iron deficiency is a major global public health problem, common in adolescence, in menstruating women and in elderly, and particularly evident in developing countries. In addition, several diseases cause iron deficiency, including intestinal parasite infections, malaria, gastric and duodenal ulcers, gastrointestinal cancer and rare mutations in genes encoding ion transporters^1, 2^. Iron deficiency can be efficaciously controlled by iron supplementation or food fortification. Unfortunately, the water-soluble and bioavailable ferrous sulphate, fumarate or gluconate, that are currently used therapeutically, affect the gastrointestinal tract with significant side effects such as constipation, diarrhoea and nausea^3^; moreover, iron salts cause unacceptable changes in the colour and taste of foods^4^. In contrast, poorly water-soluble compounds cause fewer sensory changes, but present limited bioavailability. Recently, the use of NPs in iron supplementation and fortification has been suggested since their use may combine high bioavailability, good product stability, limited side effects and absence of changes of taste and colour of the fortified foods^5–8^. Moreover, *in vitro* and *in vivo* experiments have shown that iron NPs can be considered safe^9, 10^. Nanoformulations have also found their way into the fortification of animal feeds^11–13^. Ferritin, which is well absorbed^14^, is itself composed of an iron oxide nanocore surrounded by a protein shell and, recently, a nanoparticulate mimetic of the ferritin core was proposed as a potentially side effect-free form of cheap supplemental iron^15^.

In the gut lumen, dietary iron is present as heme and non-heme (inorganic) iron; inorganic iron can be found in two oxidation states: ferrous (Fe^2+^) iron and, predominantly, as ferric (Fe^3+^) iron. Ferric iron is reduced to ferrous iron by duodenal cytochrome b, an enzyme present on the plasma membrane of the enterocyte brush border; Fe^2+^ is eventually transported into the cytoplasm by means of the divalent metal transporter DMT1^1, 16^ Nanoparticulate iron might follow a different route to access enterocyte cytoplasm. Powell and colleagues^15, 17–19^ have suggested that nanoparticulate iron, like ferritin^20^, enters cells by endocytosis reaching the cytoplasm by lysosomal escape. Endocytosis is, indeed, the canonical mechanism by which NPs are known to be up-taken^21^, but it might not be the only one. The work on ferritin core mimetics focused on highly disperse NPs with an outer layer of charged short-chain carboxylates^15^, however, poorly charged or ‘naked’ (i.e. lacking an outer coating) NPs may behave differently. In effect, it has been recently postulated that some metal NPs might also cross the plasma membrane by a non-endocytotic pathway^22–28^ gaining a direct access to the cytoplasm. Indeed, we have recently confirmed this pathway for cobalt oxide NPs. This pathway, which involves perforation of the lipid bilayer, is usually poorly considered and challenges the idea of non-permeability of membranes to hydrophilic molecules or supramolecular structures.

To verify this possibility, we have studied commercially available iron based NPs that do not comprise a dispersing outer layer. We have characterized their size and surface charge and, through a recently developed protocol, we have followed the cytoplasmic concentration dynamics of iron, demonstrating that iron oxide NPs, but not zerovalent iron NPs, were up-taken. We have also shown that the NP crossing of the plasma membrane is accompanied by a transient increase of membrane conductance, but this crossing did not occur when the iron oxide NPs were aggregated or surrounded by a protein corona.

## Results and Discussion

### Calcein and *Xenopus laevis* oocytes for monitoring intracellular iron increase

As a preliminary step, we have tested the ability of Calcein to detect intracellular iron concentration increases in fully-grown *Xenopus laevis* oocytes. Fully-grown oocytes are large cells arrested at prophase of meiosis I, which provide a simple system for the study of membrane transport. Calcein is a divalent metal ion chelating fluorochrome whose fluorescence, at physiological pH, is strongly quenched by cobalt, nickel, copper, iron and manganese divalent cations.

**Figure 1:**
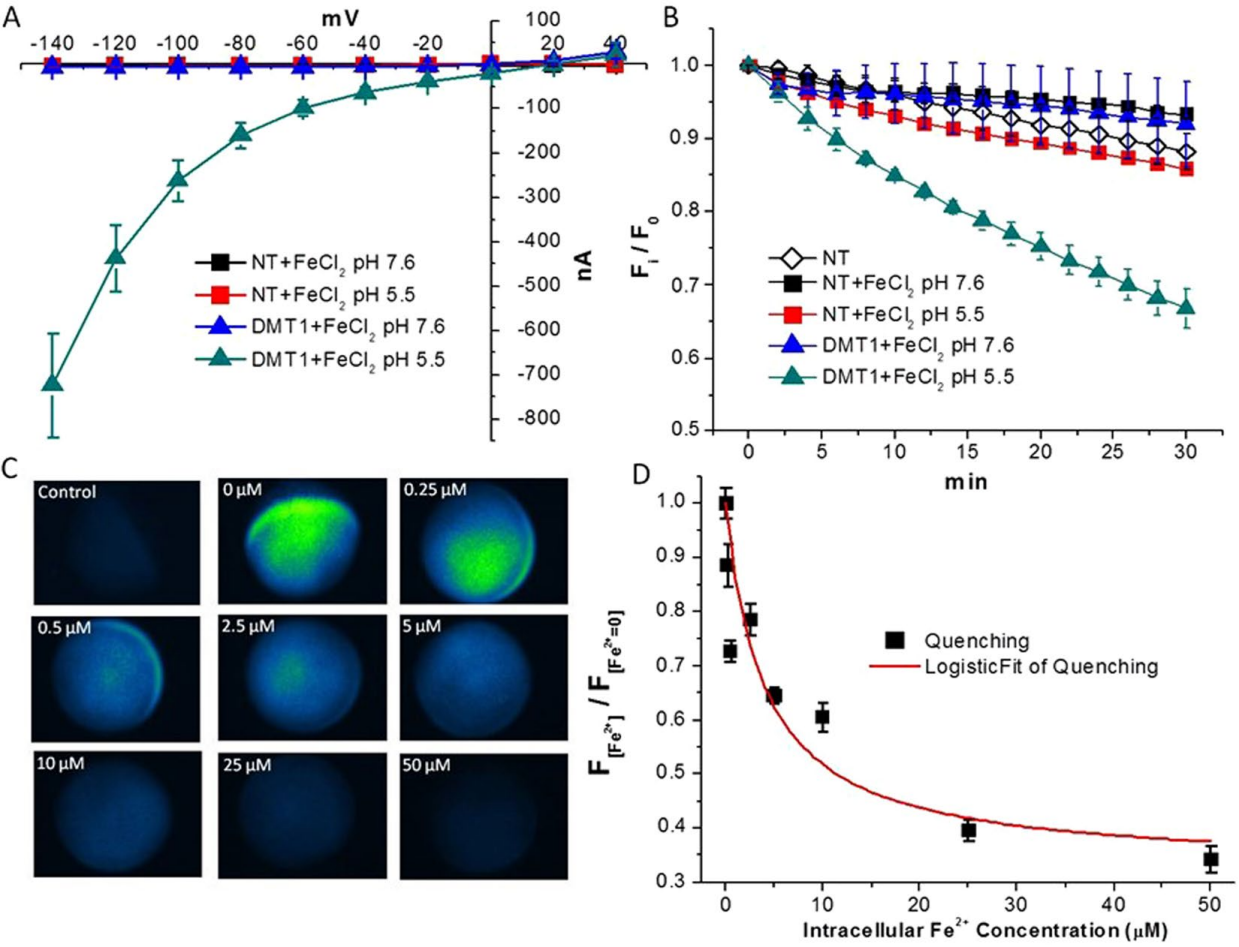
Calcein as iron detector. (**A**) Current-voltage (I-V) relationships of Non-Transfected (NT) oocytes and DMT1-expressing oocytes at pH 5.5 and 7.6; note that only DMT1-transfected oocytes at pH 5.5 are able to transport iron ions causing large inward currents. (**B**) Calcein quenching is plotted versus time as the ratio of the fluorescence (F_I_) and the fluorescence recorded at time 0 (F_0_); again only DMT1-transfected oocytes at pH 5.5 are able to transport iron ions causing the increase of their intracellular concentration. (**C**) Representative images of oocytes injected with Calcein and increasing amount of iron ions to obtain the standard curve of panel D. (**D**) The mean values of fluorescence, normalized to the mean values of fluorescence measured in oocytes injected with Calcein alone are plotted versus intracellular iron concentration; (from 60 to 80 from 3 batches for each concentration) of oocytes for each iron concentrations; data were fitted with a logistic curve (K_0.5_ = 3.97 ± 2.09 µM).

To this aim, we have prepared transfected *Xenopus* oocytes expressing the Divalent Metal ion Transporter 1 from rat (rDMT1). This membrane protein is a proton dependent transporter of divalent metal ions such as Fe^2+^, Mn^2+^, Co^2+^, Ni^2+^ and Cd^2+^ ^29–32^. We have performed voltage-clamp experiments in transfected (i.e., injected with DMT1 cRNA) and in non-transfected (control) oocytes exposed to FeCl_2_, both at pH 5.5 and 7.6. We have calculated transport currents as the difference between the currents recorded in the presence and in the absence of 100 µM FeCl_2_ in the external solution. As shown by the current-voltage (I-V) relationship (Fig. 1A), no transport currents were recorded in non-transfected (control) oocytes when exposed to iron ions confirming the absence of endogenous electrogenic Fe^2+^ transporters in the oocyte plasma membrane. Conversely, in rDMT1 transfected oocytes, at pH 5.5, but not at pH 7.6, the presence of FeCl_2_ in the extracellular solution elicited large inward currents at negative potentials. This indicates that, at pH 5.5, a transmembrane transport of iron ions occurs in rDMT1 transfected oocytes.

We repeated the same experimental plan in oocytes previously injected with Calcein (25 µM) to demonstrate that Calcein was able to detect an intracellular increase of Fe^2+^ concentration. As shown in Fig. 1B, we monitored the reduction of Calcein fluorescence for 30 min in transfected and in non-transfected oocytes exposed to 100 µM FeCl_2_ at pH 5.5 and 7.6. The results clearly show that a significant quenching of the fluorescence occurred only in DMT1 expressing oocytes at pH 5.5. It has to be noted that in DMT1 expressing oocytes at pH 7.6 no current (Fig. 1A) and no Calcein quenching (Fig. 1B) was recorded, according to the transporter pH dependence. These experiments, taken together, demonstrate that Calcein can detect an increase in the concentration of intracellular iron ions, and that oocytes are devoid of endogenous iron transporters.

To define the sensitivity of the method, we have injected oocytes with a fixed amount of Calcein together with increasing amounts of FeCl_2_. We have then measured the residual fluorescence for each iron concentration and we have recorded a dose dependent reduction of the emission values (Fig. 1C) that we have fitted with a logistic equation (Fig. 1D). Given an oocyte volume of about 1 µl, the K_0,5_ resulted 3.97 ± 2.09 (SE) µM. By means of this curve, we can estimate the concentrations of iron ions in the cytoplasm of *Xenopus* oocytes. Indeed, Fig. 1B shows that DMT1 transfected oocytes reach, 30 min after exposure to FeCl_2_, quenching values corresponding to concentrations of about the 5 µM.

After 30 min of exposure of *Xenopus* oocytes to radioactive iron, Marciani and colleagues^33^ reported concentrations slightly higher. Their experimental approach based on radioactive ^55^Fe^2+^, however, considered the total amount of iron that entered the cytoplasm through DMT1. Since a large amount of iron might become protein-bound, they estimated the total concentration of the iron ions.

### Iron oxide NPs cross the plasma membrane of *Xenopus laevis* oocytes

**Figure 2:**
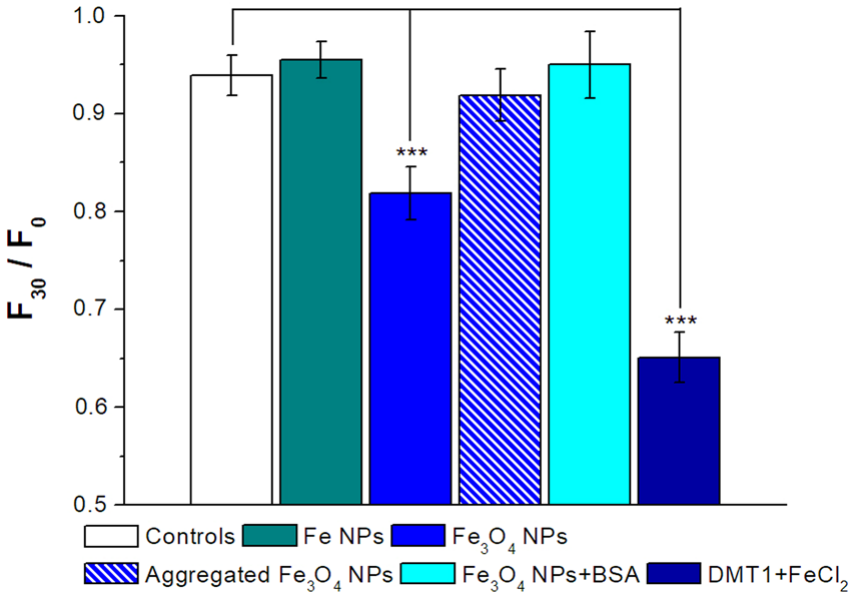
Iron oxide NPs induce intracellular iron increase. Histogram of the means of the F_30_/F_0_ values calculated as the fluorescence values at time 30 min normalized for the fluorescence values at time 0. Fe_3_O_4_ NPs (blue column) caused a statistically significant quenching of Calcein compared to the controls, i.e., Calcein injected oocytes exposed to external control solution at pH 7.6 (white column). Conversely, Fe NPs (green column), aggregated Fe_3_O_4_ NPs (blue barred column) and BSA modified Fe_3_O_4_ NPs (cyan column) did not induce quenching; rDMT1 transfected oocytes exposed to iron chloride were used as positive controls (dark blue column). Bars represent ± SEM; from 10 to 40 oocytes for each column deriving from 2 to 8 batches were used. Statistical analysis was performed with One-way ANOVA and orthogonal comparisons with Holm-Bonferroni post hoc test (***p < 0.005).

After having verified that we were able to detect an increase of iron ions in the cytoplasm of *Xenopus* oocytes that were filled with Calcein, we have used them to reveal the possible permeation of iron NPs inside the cell. To this aim, we have chosen iron NPs in two different forms, zerovalent (Fe) and oxide (Fe_3_O_4_). Similarly to other metal NPs^10, 21, 34^, iron^35^ and iron oxide^36, 37^ NPs undergo dissolution releasing iron ions that, as we have here demonstrated, can be detected by Calcein quenching. As such, we exposed Calcein-filled oocytes to iron NPs. In oocytes obtained from different batches, iron oxide NPs constantly induced a quenching of Calcein fluorescence (Fig. 2, blue column). This fluorescence decrease, although lower than that occurring in rDMT1 expressing oocytes exposed to 100 µM FeCl_2_ (Fig. 2, dark blue column), was significantly greater than that occurring in non-transfected oocytes either exposed (Fig. 1B, black squares) or not exposed (Fig. 1B, white diamond and Fig. 2, white bar) to FeCl_2_. These results suggest that Fe_3_O_4_ NPs interact with the plasma membrane of the oocyte, cross it and, once in the cytoplasm, dissolve causing the observed Calcein quenching. Zerovalent iron NPs, instead, did not cause a reduction of fluorescence (Fig. 2, dark cyan column) suggesting that, in this case, NPs do not cross the plasma membrane of the oocyte.

**Figure 3:**
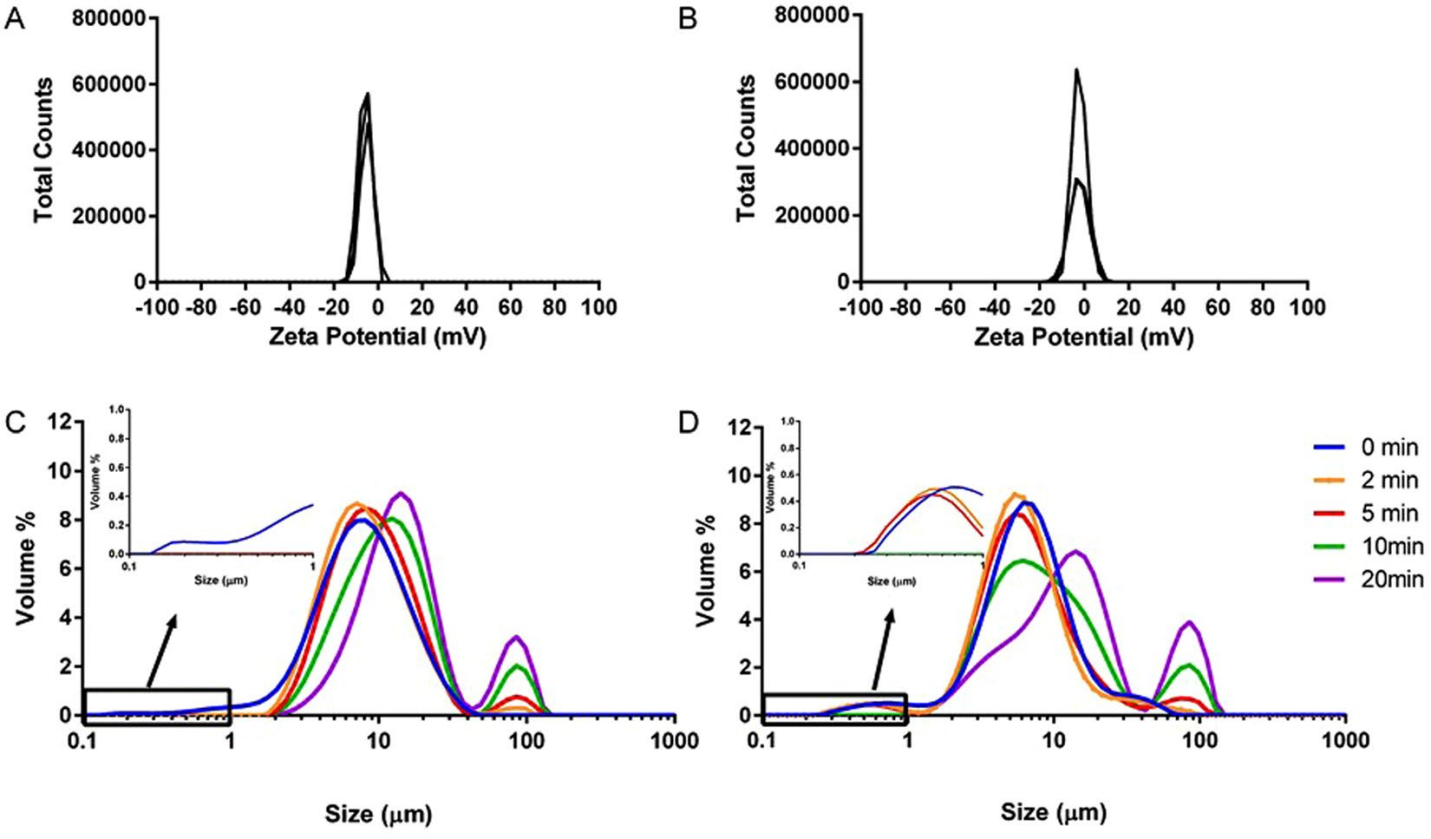
﻿Charge and hydrodynamic size of zerovalent iron (Fe^0^) (A and C) and iron oxide (Fe_3_O_4_) (B and D) NPs in external control solution (0.1 mg/mL). Zeta potential (top graphs) was determined by Laser Doppler Micro-electrophoresis as −5.0 ± 0.2 mV and −2.2 ± 0.1 mV for zerovalent iron and iron oxide NPs, respectively (N = 3). Hydrodynamic size was determined over time by Static Light Scattering (bottom graphs).

In a previous paper, we attributed the different biological behaviour (capacity to cross cell membranes) between metal oxide and zerovalent metal NPs to the chemical and physical characteristics of their surfaces. To better understand the causes of these differences, in this paper, we have measured z-potentials and described the aggregation dynamics of both NPs. The z-potentials, as determined by Laser Doppler Micro-electrophoresis (Fig. 3, top), were −5.0 ± 0.2 mV and −2.2 ± 0.1 mV for zerovalent iron and iron oxide NPs, respectively. Thus, both types of NPs are poorly charged when suspended in external control solution and this does not explain the difference in capacity to cross cell membranes, but explains why both NPs rapidly aggregate into micronized assemblies when added to media (Fig. 3, bottom) despite prior ultrasonication. Indeed, z-potentials above 30 mV or below −30 mV are generally required to maintain NPs disperse in solution through electrostatic repulsion. However, the rate of aggregation, as determined by Static Light Scattering (Fig. 3, bottom), differed between zerovalent NPs, which aggregated very rapidly, and iron oxide NPs, which retained a sub-micron population for at least 5 min (Fig. 3, insets of bottom graphs). We think that the NPs that constitute this sub-micron population are those that can cross the plasma membrane possibly because these remained below a not yet determined upper particle size limit for plasma permeation. Physical penetration of lipid bilayer membranes by NPs has been demonstrated to possibly occur by dissipative particle dynamics simulations for single NPs and for very small clusters. Aggregated NPs, instead, are likely too large to penetrate the lipid bilayer^28^. Indeed, when the oocytes were exposed to aggregated NPs, we did not record Calcein quenching (Fig. 2, blue barred column).

### NPs do not impair oocyte plasma membrane integrity, but open a transient conductance

To learn more about the interactions of NPs with the plasma membrane of the oocytes, we have followed possible changes of its integrity due to exposure to Fe_3_O_4_ NPs by Two Electrodes Voltage Clamp (TEVC). With a similar approach, Bernareggi and colleagues^38^ have recently evaluated the effects of asbestos fibres on *Xenopus* oocyte membrane.

**Figure 4:**
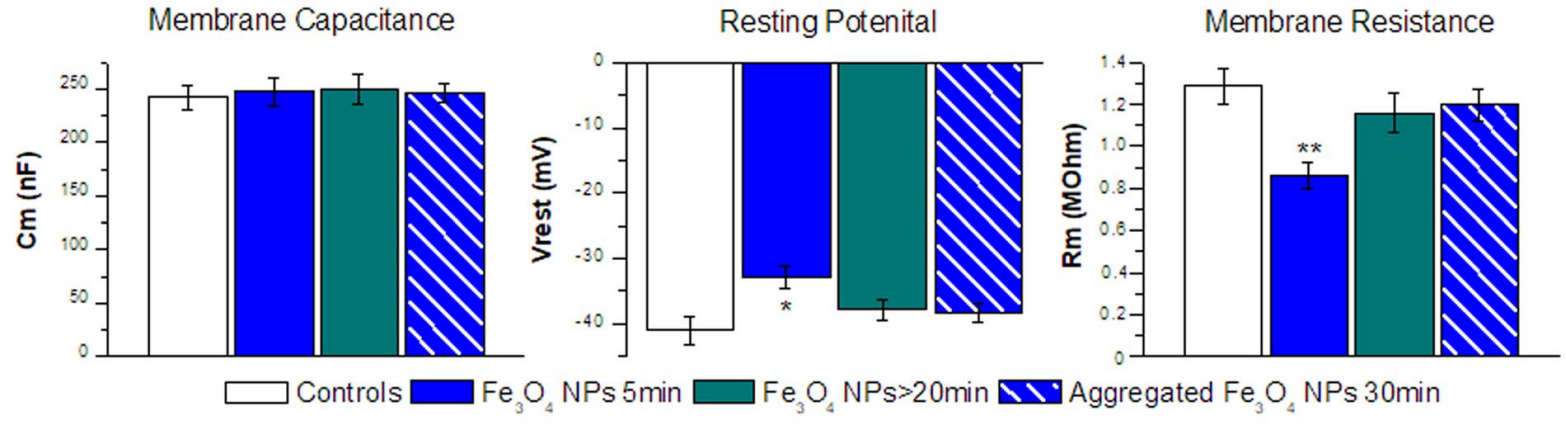
Effects of Fe_3_O_4_ NPs on oocyte membrane electrical properties. Mean values of membrane electrical parameters registered from oocytes exposed to control conditions (white columns), to a Fe_3_O_4_ NP suspension for 5 min (blue columns) or for more than 20 min (green columns), and to NPs that after sonication, were left undisturbed for 30 min (blue barred columns). Membrane capacitance of control and treated oocytes showed no significant differences at any incubation times. Resting potential and Membrane resistance significantly diminishes in oocytes treated 5 min with Fe_3_O_4_ NPs compared to their controls; the difference disappears in oocytes treated for 20 min and in oocyte treated with aggregated NPS. Bars represent mean ± SEM; 14 to 30 oocytes from 2 oocyte batches were used. Statistical analysis was performed with one-way ANOVA and orthogonal comparisons with Holm-Bonferroni post hoc test (*p < 0.05–**p < 0.01).

We have measured membrane capacitance, resting potential and resistance during a 30 min period of exposure to Fe_3_O_4_ NPs. Since we have noticed that the alterations of membrane parameters occur within the first 5 min, we have decided to perform the electrophysiological measurements after 5 and 20 min of exposure; we have also exposed oocytes to NPs that, after sonication, were left undisturbed for 30 min. A statistically significant decrease of membrane resistance, as shown in Fig. 4, occurs only in oocytes exposed for 5 min and disappears completely in oocytes exposed for 20 min. A slight decrease of resting potential, statistically significant, accompanied the decrease of membrane resistance. Membrane capacitance, instead, remained unaffected by Fe_3_O_4_ NPs treatment.

The decrease of membrane resistance can be attributed to the opening of a small transient conductance. On the other hand, the constancy of membrane capacitance is an indication that no large endocytosis phenomena occur after exposure to iron oxide NPs^39^, while the small reduction of the resting potential proves that the membrane has maintained its integrity^40^, notwithstanding a small increase of ion permeability.

**Figure 5:**
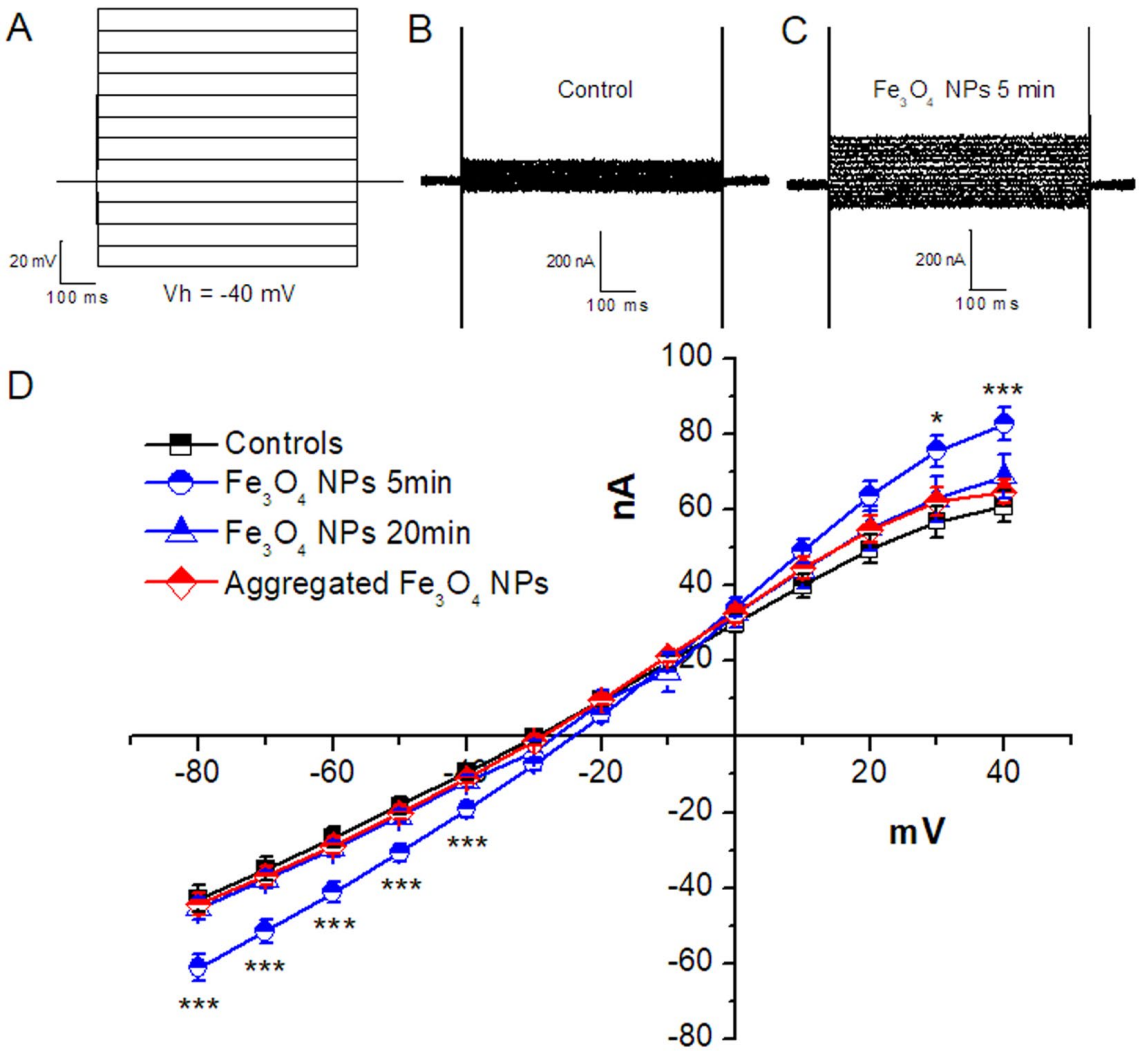
Membrane currents recorded in oocyte exposed or not to Fe_3_O_4_ NPs. (**A**) Voltage Protocol: from holding potential of −40 mV, 10 mV steps of 1 s were applied from −80 mV to +50 mV. Representative traces recorded from oocytes not exposed (**B**) or (**C**) exposed for 5 min to Fe_3_O_4_ NPs. In D, the averages of steady state membrane currents are reported as I-V relationships. After 5 min of NP exposure (blue circles), a slight, but statistically significant current increase is recorded at the curve extremes; after 20 min (blue triangles), no more statistically significant differences can be appreciated. Aggregated NP treatment generated in oocytes currents (red diamonds) almost overlapping with controls (black squares) and 20 min treatment currents, and that is an indication of the inefficacy of this treatment (*p < 0.05, ***p < 0.005; one-way ANOVA, Holm-Bonferroni post hoc orthogonal comparison; 14 to 30 oocytes from 2 batches).

To investigate further this transient conductance, we have performed a protocol to obtain I-V curves. Figure 5A shows representative current traces recorded after imposing voltage steps in a control oocyte and in an oocyte exposed to Fe_3_O_4_ NPs for 5 min. We have calculated the average currents at each potential and reported these data in the I-V curves of Fig. 5D. At potentials more negative than −40 mV and more positive than +30 mV, a slight increase of the currents obtained in exposed oocytes, compared to those in the control condition, is evident. This current increase is statistically significant only in oocytes tested within 5 min of the NPs treatment and it tends to disappear in oocytes exposed for more than 20 min and is not present in oocytes exposed to NPs that, after sonication, were left undisturbed for 30 min. The I-V curves confirm the onset of a conductance caused by the exposure to Fe_3_O_4_ NPs for 5 min and its transient nature.

### Protein corona impedes plasma membrane crossing and the opening of the transient conductance

**Figure 6:**
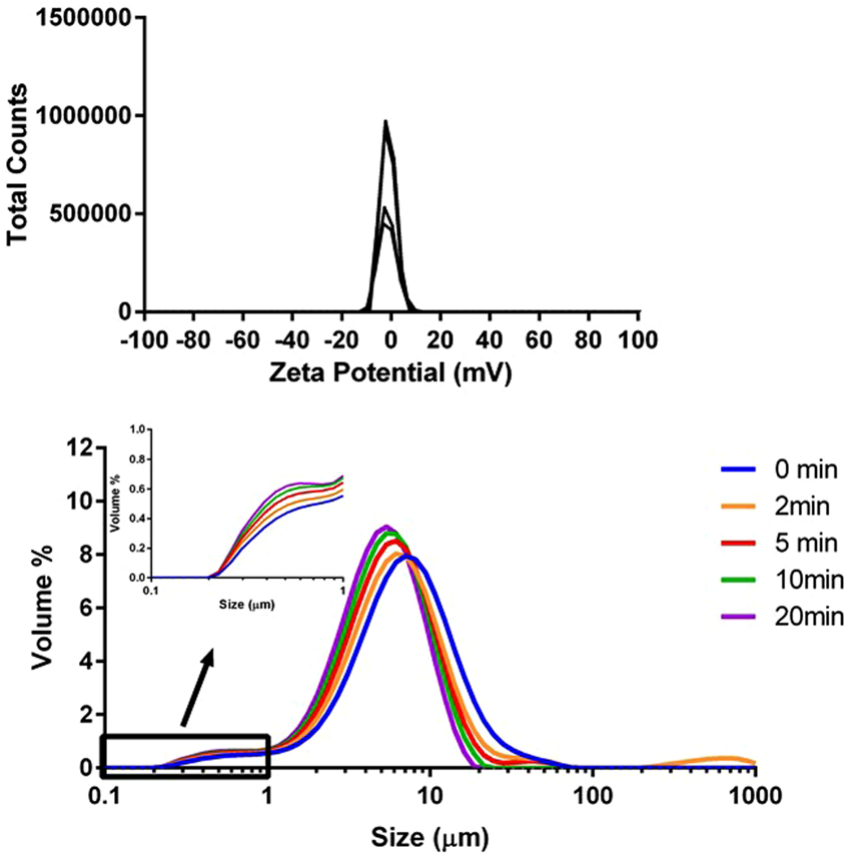
Zeta potential distribution (top) and particle size distribution over time (bottom) of a suspension of Fe_3_O_4_ NPs (0.1 mg/mL) in external control solution with 1 mg/mL BSA. Zeta potential was determined by Laser Doppler Micro-electrophoresis as −1.3 ± 0.2 mV (N = 4).

To verify our hypothesis concerning the role of the interactions between NPs and plasma membrane, we tested uptake of Fe_3_O_4_ NPs in bovine serum albumin (BSA) containing media. These molecules create a coating around the NPs, referred to as protein corona. This corona is highly dynamic and its formation and stability depends on NP characteristics and environmental variables^41^. BSA is negatively charged in water, but not in this media (Supp Figure S1) presumably due to the presence of positively charged calcium and magnesium ions. Consequently, the addition of BSA did not alter the surface charge of the iron oxide NPs (zeta potential was −1.3 ± 0.2 mV; Fig. 6 top). However, the addition of BSA did increase dispersibility as determined by the presence of the sub-micron fraction for the duration of the assay (Fig. 6). As shown above, plasma membrane crossing occurs in the presence of disperse colloids; however, exposure to BSA-coated Fe_3_O_4_ NPs did not cause fluorescence quenching (Fig. 2, cyan column). Therefore, protein corona prevents or significantly reduces the interactions between NPs and the oocyte plasma membrane blocking or limiting the passage of NPs into the cytoplasm. These findings are also in agreement with experiments where the interaction of cationic polystyrene NPs with artificial lipid bilayers were eliminated with serum proteins^42^.

**Figure 7:**
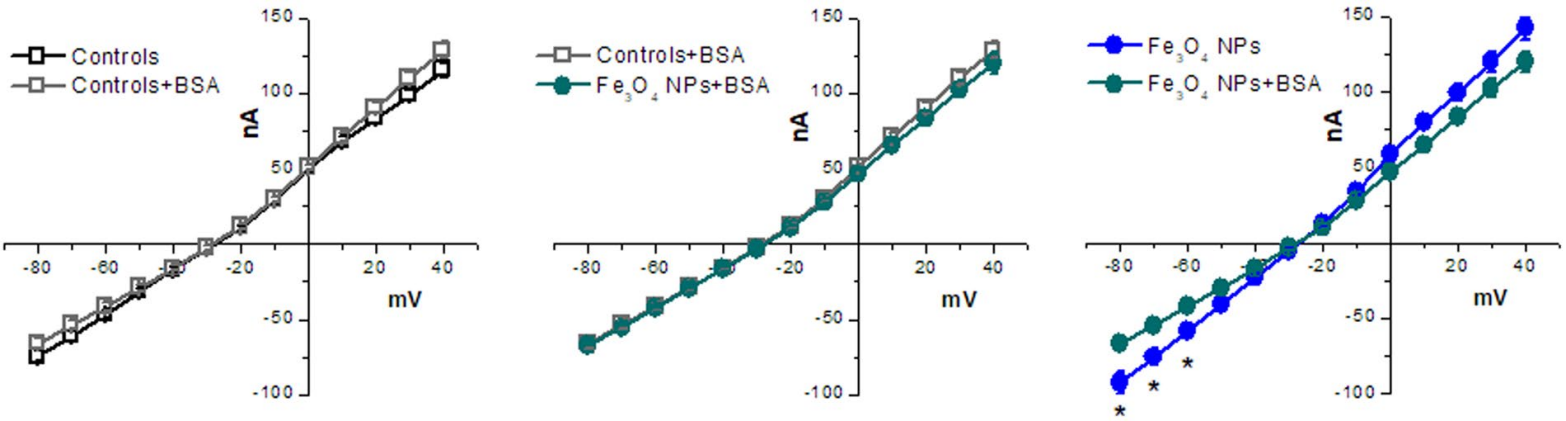
Protein corona affects NP capacity to elicit membrane currents. I-V relationships of control oocytes and oocytes treated for 5 min with BSA are compared in the left panel and no statistically significant differences are present, indicating that BSA does not affect per se membrane resistance. In the central panel, it is shown that Fe_3_O_4_ NPs that were coated with a BSA corona do not cause a decrease of the membrane resistance suspension. In the right panel, the I-V relationships of oocytes treated for 5 min with Fe_3_O_4_ NPs and BSA coated Fe_3_O_4_ NPs are compared. Their difference is statistically significant, indicating that the presence of a protein corona around Fe_3_O_4_ NPs diminishes their capacity to elicit membrane currents. Statistical analysis was performed with one-way ANOVA and orthogonal comparisons with Holm-Bonferroni post hoc test (*p < 0.05); 27 to 61 oocytes for each condition from 3 to 4 batches were used.

To confirm this hypothesis, we have performed TEVC experiments to obtain I-V curves (Fig. 7). After having verified that BSA *per se* does not affect membrane conductance, we have compared oocytes exposed to BSA-modified Fe_3_O_4_ NPs with unexposed oocytes, and to oocytes exposed to Fe_3_O_4_ NPs. These experiments suggest that, when NPs are surrounded by a protein corona, they become unable to modify membrane resistance.

## Conclusions

In conclusion, we have added further evidence to the possibility of cytomembranes being permeable to uncoated NPs. Endocytosis is the typical mechanism cells use to uptake NPs that, in this way, gain access to the endosomal compartment. Conversely, by directly crossing lipid bilayers, NPs enter cytoplasm and other cellular compartments. Moreover, we have shown that the penetration by NPs of the lipid bilayer opens a transient conductance that does not impair plasma membrane integrity.

Whether we should favour NP presence in the cytoplasm or avoid it, we have to understand the mechanisms that regulate their uptake and consider how physicochemical properties of NPs impact on their fate. In this work, we have also learned that, unlike iron oxide NPs, zerovalent iron NPs do not cross plasma membranes. We have explained this different behaviour with the very fast aggregation rate of zerovalent iron NPs. In addition, we have shown that the crossing of metal oxide NPs can be impeded by a protein corona. Indeed, proteins are abundant in the tissue fluids, but NPs could be functionalized to become corona-free^43^, thus potentially enabling the non-endocytic pathway. For example, superparamagnetic iron oxide NPs can be induced to rotate around their axes by a remote magnetic field^44^ and have been used to kill cancer cells through mechanical rupture^45^. Understanding the mechanisms that regulate NP uptake or their interactions with the cellular membrane could help in potentiating the anticancer properties of such NPs.

We also understand that the conditions iron NPs find in the gastrointestinal tract greatly differ from those of the simple model system we have used. In particular, the intestinal millieu comprises a diverse group of soluble species, ranging from small carboxylates throught bile acids to high molecular weight mucins^46–48^, many of which may interact with the surface of uncoated NPs and, in doing so, may alter their behaviour. This now merits assessment in future studies using conditions that are representative of the complex gastrointestinal environment.

## Materials and Methods

### Solutions

ND96 solution had the following composition (in mM): NaCl 96, KCl 2, CaCl_2_ 1.8, MgCl_2_ 1, HEPES 5, pH 7.6; NDE solution was composed of ND96 plus 2.5 mM pyruvate and 50 µg/mL Gentamycin sulphate; external control solution contained (in mM): NaCl 98, MgCl_2_ 1, CaCl_2_ 1.8, HEPES or MES 5, pH 7.6 or 5.5; intracellular solution contained (in mM): KCl 130, NaCl 4, MgCl_2_ 1.6, EGTA 5, HEPES 10, glucose 5, pH 7.6. The final pH values of 5.5 or 7.6 were adjusted with HCl and NaOH.

### Oocytes collection and preparation

Oocytes were obtained from adult *Xenopus laevis* females. Animals were anaesthetised in 0.1% (w/v) MS222 (tricaine methansulfonate) solution in tap water; after carefully cleaning the frog abdomen with an antiseptic agent (Povidone-iodine 10%)^49^, laparotomy was performed and portions of the ovary were collected. The oocytes were treated with 0.5 mg/mL collagenase (Sigma Type IA) in ND96 calcium free for at least 30 min at 18 °C. Healthy and fully grown oocytes were selected and stored at 18 °C in NDE solution^50^. The oocytes to be transfected with the cRNA coding for rDMT1 were injected with 25 ng of cRNA in 50 nL of water, the day after the removal, using a manual microinjection system (Drummond Scientific Company, Broomall, PA) and incubated at 18 °C for 3–4 days before electrophysiological or fluorescence experiments. The experimental protocol was approved locally by the Committee of the “Organismo Preposto al Benessere degli Animali” of the University of Insubria (OPBA-permit #02_15) and nationally by Ministero della Salute (permit nr. 1011/2015).

### NP preparation and incubation conditions

Zerovalent (Fe, 25 nm, IOLITEC, Salzstrasse 184, D-74076 Heilbronn) and oxide (Fe_3_O_4_, <50 nm TEM determined, Sigma-Aldrich) iron NPs were prepared as 10 mg/mL stock suspensions in deionised water and sonicated before addition of 10 μL to the test chamber (4 wells plate) containing 990 μL of external control solution (pH 7.6). Oocytes were then added to the chamber.

For electrophysiological experiments, the oocytes were transferred to the TEVC recording chamber within 5 min or after 20 min from the beginning of the exposure. For the fluorescence assay, recordings start immediately after oocytes addition to the test chamber. Treatments with the aggregated NPs were performed adding oocytes to the suspension of NPs that were let to aggregate in the test chamber for 30 min after sonication.

All experiments were carried out at room temperature.

### Fluorescence Assay

Oocytes were injected with a 50 nL drop of intracellular solution containing 25 µM Calcein. In dose-response evaluation experiments (Fig. 1C and D), solutions containing Calcein and increasing amounts of FeCl_2_ were co-injected in oocytes. Effective intracellular metal concentrations were calculated using the nominal oocyte volume of 1 µL^51^. Images were acquired for every oocyte at fixed acquisition parameters, calculated on control oocytes.

In experiments with NPs, images of single oocytes were acquired every 2 min for 30 min with a fluorescence microscope (AxioVert 200, Carl Zeiss with a 4x objective, COLIBRI fluorescence filters, 470 nm excitation - 515 to 565 nm emission) equipped with CCD camera (Axiocam ICM1, Carl Zeiss).

### Electrophysiology

The two-electrode voltage clamp was performed with an Oocyte Clamp OC-725B (Warner Instruments, Hamden, CT, USA) that was controlled by Clampex 10.2 (Molecular Devices, Sunnyvale, CA, USA, www.moleculardevices.com). Intracellular glass microelectrodes, filled with 3 M KCl, had tip resistances in the 0.5–4 MΩ range. Agar bridges (3% agar in 3 M KCl) connected the bath electrodes to the experimental chamber. The holding potential applied (V_h_) was −40 mV for all the experiments performed. The I-V curves of Fig. 1A were obtained applying 20 mV steps of 200 ms from −140 to +40 mV.

Oocytes were transferred in the recording chamber (Warner- RC-1Z) (warneronline.com) and impaled with microelectrodes; to recover from possible damage they were left for 2 min in a continuous solution flux. Only oocytes with resting potential equal or lower than −20 mV were used for the experiments. The number of discarded oocytes was not significantly different between treated and controls.

The capacitance and resistance values that were reported in Fig. 4 were obtained applying 10 mV steps of 20 ms every 200 ms in voltage clamp conditions. The protocol for the I-V curves of Fig. 1 was previously described^52^ and for Figs 5 and 7 is shown in Fig. 5A (10 mV steps of 750 ms from −80 to +40 mV).

### Data analysis

Data were analysed using Clampfit 10.2 software (Molecular Devices, Sunnyvale, CA, USA, www.moleculardevices.com) while OriginPro 8.0 (OriginLab Corp., Northampton, MA, USA, www.originlab.com) was used for statistics and figure preparation. For the Voltage Step protocol, current values were measured for every voltage step at the steady state condition; mean values at every voltage were calculated and plotted.

Fluorescence decay images were analysed with ImageJ (Rasband, W.S., ImageJ, U. S. National Institutes of Health, Bethesda, Maryland, USA, http://imagej.nih.gov/ij/, 1997–2015). For F_30_/F_0_ quantification, the fluorescence intensity at time 0 (F_0_) and at time 30 min (F_t_) was calculated in the entire area of the oocyte. In dose-response experiments, images were analysed calculating fluorescence intensity on the entire area of oocytes and normalized to the control oocytes. Mean values were calculated for every condition and used to determine through a non-linear fitting (Logistic Fitting, OriginPro8) the K_0.5_, relating residual fluorescence to metal concentration.

### Static light scattering (SLS)

SLS was performed on a Mastersizer 2000 with a Hydro 2000µP Micro Precision sample dispersion unit (Malvern Instruments Limited). Baseline correction was carried out with fresh external control solution (prepared as described above). Next, 200 µL of 10 mg/mL stock suspension was added to the 20 mL dispersion cell to achieve a final concentration of 0.1 mg/mL (as per Oocyte assays). The dispersion unit was run at 1000 rpm and care was taken to prevent bubble formation. The stock suspension was carefully sonicated prior to introduction to the dispersion cell, after which data acquisition was immediately initiated. Size measurements then were carried out over a period of 60 min (refractive index: 2.42; absorption 1.0; dispersant refractive index: 1.33).

### Zeta potential

The zeta potential of suspensions of iron NPs was determined by Laser Doppler Micro-electrophoresis (Zetasizer NanoZS, Malvern Instruments Ltd) using disposable folded capillary cells (DTS1070). Measurements (N = 3) were carried out using the diffusion barrier as per instrument manufacturer instructions. Briefly, the zeta cell was filled with 100 mM NaCl solution and then 100 µL sample (0.1 mg/mL suspension of NPs in external control solution) was gently injected to the bottom of the cell with a gel-loading tip. Electrophoretic mobility of particles was converted into zeta potentials by Dispersion Technology Software 7.11 using the Smoluchowski approximation, and a viscosity of 0.8872 cP and dielectric constant of 78.5 for the dispersant.

## Electronic supplementary material

supplementary info S1
