# Supplementary material for: Iron oxide nanoparticles can cross plasma membranes
Source: Sci Rep. 2017 Sep 12;7:11413. doi: 10.1038/s41598-017-11535-z (PMC5595914; doi:10.1038/s41598-017-11535-z)
Supplement: Supplementary file 1 — supplementary info S1 [file 41598_2017_11535_MOESM1_ESM.doc]

**Supplementary information**

Article: Iron oxide nanoparticles can cross plasma membranes

Daniele Zanella^1^, Elena Bossi^1,2^*, Rosalba Gornati^1,2^, Carlos Bastos^3^, Nuno Faria^3^ and Giovanni Bernardini^1,2^

^1^Department of Biotechnology and Life Sciences, University of Insubria; Via Dunant 3, I-21100 Varese, Italy

^2^Interuniversity Center “The Protein Factory”, Politecnico di Milano and Università dell'Insubria, Via Mancinelli 7, I-20131 Milan, Italy

^3^Department of Veterinary Medicine, University of Cambridge, Madingley Road, Cambridge CB3 OES, UK

*Corresponding Author: Elena Bossi, e-mail: elena.bossi@uninsubria.it

**Supplementary figure**

***Figure S1.*** *Zeta potential distribution of a BSA solution (40 mg/mL) in external control solution (Top; N=2) and in water (Bottom, N=3)*.
